# Body Image in Women During the Menopausal Transition: Associations with Self-Perceived Attractiveness in Partner Relationships

**DOI:** 10.3390/jcm15103562

**Published:** 2026-05-07

**Authors:** Agnieszka Bień, Beata Pięta, Beata Górska, Grażyna Bączek, Mariola Głowacka, Joanna Grzesik-Gąsior

**Affiliations:** 1 Chair of Obstetrics Development, Faculty of Health Sciences, Medical University of Lublin, 20-059 Lublin, Poland; agnieszka.bien@umlub.edu.pl; 2 Department of Practical Midwifery Education, Poznan University of Medical Sciences, 61-701 Poznań, Poland; 3 Independent Public Health Care Centre, Cardinal Stefan Wyszyński Regional Specialist Hospital, 20-718 Lublin, Poland; gorskabeata82@gmail.com; 4 Department of Obstetrics and Gynecology Didactics, Medical University of Warsaw, 02-091 Warsaw, Poland; grazyna.baczek@wum.edu.pl; 5 Nursing Department, Faculty of Health Sciences, Collegium Medicum, Masovian University in Płock, 09-402 Płock, Poland; m.glowacka@mazowiecka.edu.pl; 6 State University of Applied Sciences in Krosno, 38-400 Krosno, Poland; joanna.grzesik-gasior@pans.krosno.pl

**Keywords:** body image, menopausal transition, self-attractiveness, partner relationship, sexual health

## Abstract

**Background/Objectives**: The menopausal transition involves bodily, emotional, and sexual changes that may be relevant to body image. This study examined whether intrapersonal and relational dimensions of perceived self-attractiveness were associated with multidimensional body image among partnered women during the menopausal transition, after adjustment for BMI and sociodemographic factors. **Methods:** This cross-sectional study was conducted in 2024–2025 in primary health care clinics in Lublin Province, Poland, and included 474 partnered women in perimenopause or early postmenopause. Body image was assessed with the Body Esteem Scale subscales, namely Sexual Attractiveness, Weight Concern, and Physical Condition, and perceived self-attractiveness with the Scale of Assessment of Self-Attractiveness in a Relationship. Hierarchical linear regression models were adjusted for BMI, age, education, and socioeconomic conditions. **Results:** Body Acceptance was positively associated with Sexual Attractiveness (*β* = 0.193, *p* = 0.002), Weight Concern (*β* = 0.225, *p* < 0.001), and Physical Condition (*β* = 0.206, *p* = 0.001). Sexual Satisfaction was positively associated with Sexual Attractiveness (*β* = 0.299, *p* < 0.001) and Physical Condition (*β* = 0.143, *p* = 0.023). Appearance Evaluation was positively associated with Weight Concern (*β* = 0.126, *p* = 0.023). Higher BMI was negatively associated with Weight Concern (*β* = −0.356, *p* < 0.001) and Physical Condition (*β* = −0.209, *p* < 0.001), whereas older age was negatively associated with Sexual Attractiveness (*β* = −0.127, *p* = 0.002) and Physical Condition (*β* = −0.137, *p* = 0.001). **Conclusions:** In partnered women during the menopausal transition, body image was associated not only with BMI and age, but also with body acceptance and selected intimacy-related dimensions of perceived self-attractiveness.

## 1. Introduction

The menopausal transition is a life stage during which hormonal changes and aging processes contribute to changes in body composition and fat distribution, including increased central adiposity and loss of lean mass [1,2]. Vasomotor symptoms are common and frequently co-occur with sleep disturbances and mood changes, and sexual functioning may also change across the transition [3]. These changes may be relevant to women’s perceptions of their bodies as well as to their sense of femininity and attractiveness. Because clinicians providing menopause care, including gynecologists, midwives, and other women’s health professionals, increasingly address both somatic and psychosocial concerns in midlife, understanding factors associated with body image during this period has direct clinical relevance [4,5].

Body image is a multidimensional construct encompassing beliefs and interpretations about appearance, related emotions, and body-focused behaviors within a social context [6]. In line with multidimensional and cognitive-behavioral perspectives on body image, body-related evaluations are shaped not only by physical characteristics, but also by self-perceptions, emotions, social experiences, and interpretive processes [6]. In midlife, body evaluation often reflects not only aesthetics but also functionality and physical capability [6,7]. Body image is associated with individual factors, such as age, body mass index (BMI), health status, and personality traits, as well as with sociocultural influences, including norms regarding attractiveness [4,8]. In many Western cultures, ideals emphasizing youth, thinness, and fitness may intensify social comparison and undermine body satisfaction as women experience age-related changes [8]. Clinically, negative body image in midlife has been linked to lower well-being, avoidance of intimacy, and reduced engagement in health-promoting behaviors, underscoring its relevance for counseling in routine care [8,9]. In this context, interpersonal feedback within partner relationships may be relevant to how women evaluate their bodies during the menopausal transition.

Partner relationships provide a context in which the body may gain significance as a source of closeness and sexual satisfaction, but also as a potential target of evaluation and fear of rejection. Perceived acceptance from a significant other may be related to less negative self-evaluation, including lower body dissatisfaction. At the same time, intrapersonal dimensions, such as body acceptance and appearance evaluation, may be relevant to how women interpret external cues and integrate bodily changes during this period [10]. Accordingly, perceived self-attractiveness in partner relationships can be conceptualized as a relationship-contextualized construct that integrates intrapersonal components, such as body acceptance and appearance evaluation, with relational components, such as perceived partner acceptance and sexual satisfaction. Although related to body image, this construct is not identical to domain-specific body evaluation, which makes it clinically relevant to examine how these dimensions correspond with different aspects of body image [11].

Findings in midlife women are often less consistent than those reported in younger adults. This may reflect heterogeneity in menopausal stage, weight-change trajectories, relationship duration and quality, comorbidities, and the increasing salience of functional aspects of the body [12]. This underscores the need for analyses that consider relational and intrapersonal dimensions of self-attractiveness alongside key sociodemographic and health-related correlates.

Limited research has examined how relational and intrapersonal dimensions of perceived self-attractiveness are linked to multidimensional body image during the menopausal transition while accounting for BMI and key sociodemographic factors. This gap is clinically important because body image concerns in midlife may be relevant to well-being, intimacy, and engagement in health-promoting behaviors, yet they are often reduced in practice to weight- or symptom-centered concerns alone. Therefore, this study examined associations between perceived self-attractiveness in partner relationships and three domains of body image among partnered women during the menopausal transition, with the aim of informing more person-centered and psychosocially sensitive menopause care.

## 2. Materials and Methods

### 2.1. Study Population and Procedures

The study was conducted between January 2024 and April 2025 in several public and private primary health care clinics in Lublin Province, Poland. Women were recruited during routine clinic visits, including preventive consultations, general health assessments, and visits related to menopausal transition symptoms. Eligibility was assessed during a structured health, gynecologic, and obstetric interview covering menstrual patterns and menopausal symptoms. After receiving information about the study, participants provided written informed consent and completed the questionnaires in private.

Women who were sufficiently fluent in Polish to complete the questionnaires independently were eligible. Based on a structured interview, participants were classified as being in perimenopause (menopausal transition) or early postmenopause, defined as the first 12 months after the final menstrual period (FMP). Additional inclusion criteria were self-reported naturally occurring menopausal transition symptoms. Menopausal status was determined from interview data on bleeding patterns and climacteric symptoms, consistent with the Stages of Reproductive Aging Workshop + 10 (STRAW + 10) recommendations. Because of the cross-sectional design, classification was based on interview data only, without hormonal assessment or prospective confirmation of the FMP.

Women were excluded if they did not provide informed consent, withdrew from the study, were more than 12 months after the FMP, did not report menopausal transition symptoms, were not sufficiently fluent in Polish, or did not complete the questionnaires. No formal a priori sample size calculation was performed because this was an observational study based on a purposively recruited sample collected within a predefined study period rather than a prospectively powered design targeting a single primary parameter estimate. To provide additional context for model adequacy, we considered the final analytic sample in relation to the planned multivariable regression models. With 474 participants and nine variables included in the fully adjusted models, the sample size provided adequate sensitivity to detect small overall effects in multiple regression, assuming α = 0.05 and conventional power of 0.80. Therefore, the final analytic sample was considered sufficient for the planned analyses. Women with incomplete questionnaires were excluded from the analysis, and no item-level missing data were present among participants included in the final analytic sample.

Because the SAAR assesses perceived self-attractiveness in the context of an intimate relationship, the present analysis was restricted to women who reported being in a partner relationship.

This study was reported in accordance with the Strengthening the Reporting of Observational Studies in Epidemiology (STROBE) guidelines.

### 2.2. Data Collection

Data were collected using the Body Esteem Scale (BES), the Scale of Assessment of Self-Attractiveness in a Relationship (SAAR), and a study questionnaire. The study questionnaire collected sociodemographic data, including age, place of residence, relationship status, education, employment status, self-rated socioeconomic conditions, and parental status, as well as health-related information, including body mass index (BMI). BMI was calculated as weight in kilograms divided by height in meters squared, using self-reported height and weight.

The Body Esteem Scale (BES) was developed by Franzoi and Shields and adapted into Polish by Lipowska and Lipowski. It assesses body-related attitudes across three subscales: Sexual Attractiveness, Weight Concern, and Physical Condition. The BES comprises 35 items rated on a 5-point Likert scale, ranging from 1 (strongly negative feelings) to 5 (strongly positive feelings). Subscale scores were calculated as the sum of item responses, with higher scores indicating a more positive body evaluation. Therefore, for the Weight Concern subscale, higher scores reflect more favorable weight-related body evaluation rather than greater concern. In validation studies of the Polish version among women, internal consistency was high (Cronbach α = 0.80 for Sexual Attractiveness, 0.89 for Weight Concern, and 0.82 for Physical Condition) [13,14].

The women’s version of the Scale of Assessment of Self-Attractiveness in a Relationship (SAAR) was developed by Chybicka and Karasiewicz to assess perceived physical and sexual self-attractiveness in the context of a partner relationship. It includes four subscales, two reflecting self-evaluation (Body Acceptance and Appearance Evaluation) and two reflecting partner-related perceptions (Partner Acceptance and Sexual Satisfaction). Each subscale contains four items rated on a 5-point Likert scale, ranging from 1 (strongly disagree) to 5 (strongly agree), with higher scores indicating greater perceived self-attractiveness, partner-related acceptance, or sexual satisfaction, depending on the subscale. Reported internal consistency was acceptable (Cronbach α = 0.75 for the total scale; 0.67 for Body Acceptance, 0.74 for Appearance Evaluation, 0.71 for Partner Acceptance, and 0.70 for Sexual Satisfaction) [15].

Although related, these constructs are not identical. In the present study, body image was operationalized using the BES as domain-specific body evaluation (sexual attractiveness, weight-related evaluation, and physical condition), whereas the SAAR was used to capture perceived self-attractiveness in the context of an intimate relationship, including both intrapersonal and partner-related dimensions. Thus, SAAR Body Acceptance was treated as a relationship-contextualized dimension of perceived self-attractiveness examined in relation to BES body image domains, rather than as a component of the BES outcome itself. Nevertheless, some conceptual proximity is plausible, particularly between SAAR Body Acceptance and more global positive body evaluation, and this should be considered when interpreting associations between these measures.

To reduce potential sources of bias, questionnaires were completed in private, eligibility criteria were applied consistently during recruitment, validated instruments were used, and covariates were specified a priori.

### 2.3. Ethical Considerations

The study was conducted in accordance with the Declaration of Helsinki and Good Clinical Practice. Ethical approval was obtained from the Bioethics Committee at the Medical University of Lublin (Resolution No. KE-0254/265/12/2023, 14 December 2023).

### 2.4. Statistical Analysis

Statistical analyses were performed using Python, version 3.11.2. Categorical variables were summarized as counts and percentages, and continuous variables as mean (SD). Associations between perceived self-attractiveness in a partner relationship and body image were examined using hierarchical multiple linear regression, with separate models fitted for each Body Esteem Scale subscale. Covariates were selected a priori based on their established or plausible associations with body image in midlife and their potential to confound associations between perceived self-attractiveness in partner relationships and body image. BMI and age were included as core body-related and demographic factors, whereas education and self-rated socioeconomic conditions were included as structural indicators that may shape body-related experiences, health resources, and psychosocial context. In step 1, SAAR subscales (Body Acceptance, Appearance Evaluation, Partner Acceptance, and Sexual Satisfaction) were entered. Body mass index (BMI) was added in step 2, age in step 3, education (3 categories) in step 4, and self-rated socioeconomic conditions (satisfactory vs. unsatisfactory) in step 5, with prespecified reference categories for categorical variables. Sequential entry of covariates was used to examine the incremental contribution of body-related (BMI), demographic (age), and structural (education and socioeconomic conditions) background characteristics to the associations between SAAR dimensions and body image. Results are presented as unstandardized (B) and standardized (*β*) coefficients with 95% CIs. Model fit was evaluated using R^2^, and adjusted R^2^. Multicollinearity was assessed using variance inflation factors, with values below 5 considered not indicative of problematic collinearity. Statistical significance was set at *p* < 0.05.

## 3. Results

### 3.1. Participant Characteristics

A total of 621 women were approached during recruitment, of whom 18 were excluded because they did not meet the study eligibility criteria. The final study sample comprised 603 women, including 474 partnered women who constituted the analytic sample for the present study.

Most participants lived in the regional capital (43.88%), had higher education (49.79%), and were employed (87.55%). Most rated their socioeconomic conditions as satisfactory (76.37%) and reported having children (79.32%). Comorbidities were reported by 53.38% of participants, 74.89% reported no current tobacco and/or alcohol use, and 50.84% had normal weight (Table 1).

### 3.2. Descriptive Statistics of BES and SAAR

Descriptive statistics for the Body Esteem Scale and the Scale of Assessment of Self-Attractiveness in a Relationship subscales are presented in Table 2.

Across all regression models, VIF values were below 5, indicating no problematic multicollinearity among the variables included in the models.

### 3.3. Factors Associated with Sexual Attractiveness

Hierarchical regression results for Sexual Attractiveness are presented in Table 3. Model fit increased modestly across steps, from 22.1% to 26.2% of explained variance, indicating that the addition of BMI, age, education, and socioeconomic conditions provided only a small incremental contribution beyond the SAAR dimensions. In the fully adjusted model, Body Acceptance (*β* = 0.193, *p* = 0.002) and Sexual Satisfaction (*β* = 0.299, *p* < 0.001) were positively associated with Sexual Attractiveness, whereas age was negatively associated with Sexual Attractiveness (β = −0.127, *p* = 0.002). Appearance Evaluation, Partner Acceptance, BMI, education, and self-rated socioeconomic conditions were not significantly associated with Sexual Attractiveness in the fully adjusted model.

### 3.4. Factors Associated with Weight Concern

Table 4 presents the hierarchical regression results for Weight Concern. Model fit increased from 19.4% to 32.5% of explained variance, with the largest improvement observed after adding BMI, indicating that BMI made the strongest incremental contribution to the weight-related body image domain. In the fully adjusted model, BMI was negatively associated with Weight Concern (*β* = −0.356, *p* < 0.001), whereas Body Acceptance (*β* = 0.225, *p* < 0.001) and Appearance Evaluation (*β* = 0.126, *p* = 0.023) were positively associated with this domain. Age, Partner Acceptance, Sexual Satisfaction, education, and self-rated socioeconomic conditions were not significantly associated with Weight Concern in the fully adjusted model.

### 3.5. Factors Associated with Physical Condition

Table 5 shows the hierarchical regression results for Physical Condition. Model fit increased from 14.2% to 21.9% of explained variance, indicating a modest incremental contribution of BMI, age, education, and socioeconomic conditions beyond the SAAR dimensions. In the fully adjusted model, BMI (β = −0.209, *p* < 0.001) and age (β = −0.137, *p* = 0.001) were negatively associated with Physical Condition, whereas Body Acceptance (β = 0.206, *p* = 0.001) and Sexual Satisfaction (β = 0.143, *p* = 0.023) were positively associated with this domain. Appearance Evaluation, Partner Acceptance, education, and self-rated socioeconomic conditions were not significantly associated with Physical Condition in the fully adjusted model.

## 4. Discussion

This study contributes to the clinical literature on menopausal health by indicating that body image during the menopausal transition may follow domain-specific patterns rather than reflect a single uniform process. In the fully adjusted models, Body Acceptance was the most consistent correlate across all three body image domains, Sexual Satisfaction was relevant primarily to sexual and functional domains, and BMI was most strongly related to the weight-related domain. Clinically, this pattern suggests that body image concerns in midlife should not be approached as synonymous with body weight alone. This interpretation is consistent with broader evidence indicating that menopausal symptoms are associated with more negative body image and that sexual satisfaction remains an important dimension of well-being in postmenopausal women [3,12,16,17].

Body Acceptance was consistently related to all three body image domains. This finding may partly reflect the role of intrapersonal self-evaluation in how women appraise their bodies during the menopausal transition, but it should also be interpreted in light of the conceptual proximity between SAAR Body Acceptance and positive body evaluation. In this study, Body Acceptance was treated as a relationship-contextualized dimension of perceived self-attractiveness rather than as a BES body image outcome; nevertheless, overlap between these constructs may have contributed to the strength and consistency of the observed associations. This pattern is consistent with the view that body image in midlife may become increasingly tied to the internal integration of bodily changes rather than to appearance standards alone. Recent qualitative work suggests that women in midlife often describe tension between efforts to accept age-related bodily changes and persistent emotional discomfort related to changing appearance, bodily function, and self-perception [4,18,19].

In the present study, Sexual Satisfaction was associated with Sexual Attractiveness and Physical Condition. This pattern may reflect links between intimate comfort, agency, and positive embodied experiences on the one hand, and sexual body image and perceived physical capability on the other. However, the direction of these associations cannot be determined; more positive body image may also be related to greater sexual satisfaction. By contrast, Partner Acceptance was not significantly associated with any BES body image domain after adjustment for the other SAAR dimensions and covariates. This finding should not be interpreted as evidence that relational dynamics are unimportant for body image in midlife, but rather that the specific partner-related measure used here did not show significant associations in the fully adjusted models. Possible explanations include limited variability in the Partner Acceptance subscale, measurement constraints, or the absence of broader relationship-quality indicators such as emotional intimacy, communication, relationship duration, and overall relationship satisfaction [4,18].

Evidence in midlife women remains less consistent than in younger adults, likely because of heterogeneity in menopausal stage, weight-change trajectories, relationship characteristics, comorbidities, and the increasing salience of functional aspects of the body [19,20,21]. Recent qualitative syntheses further suggest that women’s sexual and relational experiences during the menopausal transition are shaped not only by physical symptoms, but also by relationship dynamics, communication, and broader biopsychosocial context [22,23]. The pattern observed in the present study may therefore indicate that intrapersonal and relational dimensions of perceived self-attractiveness are related to body image in different ways across domains. However, these associations should be interpreted cautiously, as the cross-sectional design precludes conclusions about temporal ordering, and partial conceptual proximity between some self-evaluative constructs may have contributed to the observed associations.

Appearance Evaluation showed a domain-specific pattern in relation to Weight Concern, but not Sexual Attractiveness or Physical Condition. This may indicate that appearance evaluation is most relevant to the body image domain that is especially sensitive to weight- and shape-related pressures and social comparison [8]. This interpretation is consistent with emerging evidence indicating that sociocultural appearance pressures and appearance comparisons remain relevant to body image concerns in women in midlife, particularly in relation to weight and shape [24]. In contrast, sexual body image in midlife may be more closely linked to Body Acceptance and the quality of intimate experiences than to appearance evaluation alone [8,25]. A recent qualitative synthesis suggests that women’s sexual experiences across the menopause continuum are shaped not only by bodily changes but also by self-perception, emotional responses, and relationship dynamics, which may help explain why Appearance Evaluation was not significantly associated with Sexual Attractiveness in the present study [22]. For clinicians, this distinction may support tailoring counseling to the specific domain of concern, such as weight- and shape-related concerns versus sexual attractiveness or perceived physical capability, rather than treating body image as a single construct.

BMI was negatively associated with Weight Concern and was also negatively associated with Physical Condition, whereas it was not significantly associated with Sexual Attractiveness. Because higher Weight Concern scores indicate more favorable weight-related body evaluation rather than greater concern, this negative association indicates that higher BMI was related to less favorable weight-related body evaluation. Similar associations between BMI, weight-related factors, and body dissatisfaction have been reported in middle-aged women [26]. Recent reviews further suggest that weight gain and changes in body composition during midlife are driven by both aging-related and menopause-related processes and may have implications not only for physical health, but also for body-related self-perception [1,4,27]. This pattern is consistent with the interpretation that weight- and shape-related factors are central to the weight-related body image domain, whereas sexual body image may be more closely linked to intrapersonal acceptance and intimate experiences than to body weight alone.

Older age was negatively associated with Sexual Attractiveness and Physical Condition, which may be related to unmeasured factors such as symptom burden, reduced energy, or functional changes during the menopausal transition [3,4]. Education and self-rated socioeconomic conditions were not significantly associated with body image in the fully adjusted models. Given the simple operationalization of these variables, these null findings should be interpreted cautiously. Because body dissatisfaction has been linked to poorer mental health outcomes and lower self-esteem, whereas more positive body image has been linked to emotional well-being, these findings support the clinical relevance of addressing psychosocial aspects of body image in women’s health care during midlife [28].

Several complementary mechanisms may help contextualize the observed associations, although these interpretations remain tentative because of the cross-sectional design. Body Acceptance may represent a psychological resource linked to self-kindness and reduced self-criticism, which may be associated with less negative interpretations of bodily changes during the menopausal transition [29]. Supportive relationship experiences may also be related to how women interpret and negotiate sociocultural pressures and social comparison during this period [8]. In the present study, this broader pattern was partly reflected in Sexual Satisfaction, which was associated with Sexual Attractiveness and Physical Condition, but not with Weight Concern. Together with previous evidence linking more favorable body image to emotional well-being and engagement in wellness behaviors, these findings are consistent with the understanding of body image in midlife within a broader biopsychosocial context rather than as a simple consequence of aging alone [5,30,31].

### 4.1. Clinical Implications

For clinicians involved in menopause care, these findings are consistent with extending routine assessment beyond somatic symptoms and body weight to include brief evaluation of body-related distress and sexual well-being. In routine visits, nonjudgmental questions may help identify women experiencing body image concerns beyond BMI and age alone, within the scope of the variables measured in this study. Examples include: “How comfortable do you feel with the changes in your body during this stage of life?”, “Have changes in your body affected how attractive or confident you feel?”, and “Has the menopausal transition affected your comfort with intimacy or sexual satisfaction?”

When concerns are identified, counseling may incorporate normalization of menopausal changes, body-neutral or function-focused reframing (e.g., strength, energy, mobility), and brief strategies that foster self-compassion and reduce self-criticism. When low sexual satisfaction co-occurs with distress about sexual attractiveness or avoidance of intimacy, referral for sexual health counseling, psychological support, or couple-based support may be considered. Lifestyle guidance, including physical activity, should be individualized and framed not only in terms of weight management, but also as supporting physical functioning, symptom coping, and overall well-being.

Overall, this approach may help clinicians address weight concerns, body comfort, perceived attractiveness, and sexual well-being as related but distinct aspects of menopause care.

### 4.2. Strengths and Limitations

This cross-sectional study included a relatively large sample of women in perimenopause and early postmenopause who reported naturally occurring menopausal symptoms. Use of validated instruments, the Body Esteem Scale and the Scale of Assessment of Self-Attractiveness in a Relationship, allowed assessment of three body image domains as well as both intrapersonal and relational dimensions of perceived self-attractiveness. The hierarchical regression approach, with adjustment for age, BMI, education, and self-rated socioeconomic conditions, helped address the possibility that the observed associations were explained solely by basic sociodemographic or weight-related factors. Recruitment in primary health care settings also supports the clinical relevance of the findings, although the sample was not intended to be population-representative.

Several limitations should be considered. First, the cross-sectional design precludes causal inference, and reverse or bidirectional relationships cannot be excluded. Second, participants were recruited purposively from primary health care settings in a single region of Poland, which may limit generalizability and introduce selection effects related to a health-seeking population. The findings may also be partly shaped by the cultural context of this clinic-based sample, including norms surrounding aging, femininity, and body evaluation, and therefore may not transfer directly to women from more diverse sociocultural or health care settings. Third, all measures were self-reported and collected from the same source, increasing the possibility of reporting bias and common method bias. In particular, BMI was derived from self-reported height and weight, which may have introduced measurement error, including possible underreporting of weight and consequent underestimation of BMI. Fourth, menopausal stage was classified using interview data on bleeding patterns and symptoms, consistent with STRAW + 10 recommendations, but without hormonal verification or prospective confirmation of the final menstrual period. This may have resulted in some misclassification, particularly near the 12-month threshold.

Another limitation is the possibility of partial conceptual proximity between SAAR Body Acceptance and body image constructs assessed with the BES. Importantly, although multicollinearity diagnostics did not indicate problematic statistical collinearity among the variables included in the regression models, this does not fully exclude theoretical closeness between these constructs. Therefore, the associations involving Body Acceptance should be interpreted with caution.

Finally, the models did not include several potentially relevant variables, such as menopausal symptom severity, depressive or anxiety symptoms, menopausal hormone therapy use, physical activity, relationship quality or duration, and partner-reported data. Their omission increases the possibility of residual confounding and limits confidence that the observed associations would remain unchanged after adjustment for these factors. Although comorbidity data were collected descriptively, they were not included in the regression models because of their broad and heterogeneous nature and because the analytic strategy was designed to focus on a parsimonious set of core background covariates specified a priori.

## 5. Conclusions

Among partnered women during the menopausal transition, Body Acceptance was the most consistent correlate of multidimensional body image, whereas Sexual Satisfaction was additionally associated with Sexual Attractiveness and Physical Condition. Overall, the data indicate that body image concerns in midlife are related not only to BMI and age, but also to psychosocial and intimacy-related dimensions of perceived self-attractiveness that may be clinically relevant in menopause care. Longitudinal and intervention studies are needed to clarify temporal pathways and to identify effective strategies to support body acceptance and sexual well-being in this population.

## Figures and Tables

**Table 1:** Characteristics of the analytic sample of partnered women (n = 474).

Characteristic		N (%)
Age, mean (SD)		50.39 (3.48)
Place of residence	Regional capital	208 (43.88)
	Other city	164 (34.60)
	Rural area	102 (21.52)
Education	Primary/vocational	37 (7.81)
	Secondary	201 (42.41)
	Higher	236 (49.79)
Employment status	Employed	415 (87.55)
	Unemployed	31 (6.54)
	On disability pension/retired	28 (5.91)
Self-rated socioeconomic conditions	Satisfactory	362 (76.37)
	Unsatisfactory	112 (23.63)
Having children	No	98 (20.68)
	Yes	376 (79.32)
Comorbidities	No	221 (46.62)
	Yes	253 (53.38)
Current tobacco and/or alcohol use	No	355 (74.89)
	Yes	119 (25.11)
BMI	Underweight (<18.5)	5 (1.05)
	Normal weight (18.5–24.9)	241 (50.84)
	Overweight (25.0–29.9)	148 (31.22)
	Obesity (≥30.0)	80 (16.88)

**Table 2:** Scores of Body Esteem Scale and Scale of Assessment of Self-Attractiveness in a Relationship in the study sample (n = 474).

Variables	M	Me	SD	Min	Max
Body Esteem Scale					
Sexual Attractiveness	44.01	44.00	8.41	22.00	65.00
Weight Concern	31.48	31.00	8.11	10.00	50.00
Physical Condition	29.82	31.00	6.68	11.00	45.00
Scale of Assessment of Self-Attractiveness in a Relationship					
Body Acceptance	2.93	3.00	0.35	1.75	4.50
Appearance Evaluation	3.11	3.00	0.38	1.75	4.75
Partner Acceptance	3.06	3.00	0.40	1.50	4.75
Sexual Satisfaction	3.03	3.00	0.42	1.00	4.50

M, mean; Me, median; SD, standard deviation; Min, minimum value; Max, maximum value.

**Table 3:** Hierarchical Linear Regression Models Examining Factors Associated with Sexual Attractiveness Scores on the Body Esteem Scale from Subscales of the Scale of Assessment of Self-Attractiveness in a Relationship and Covariates.

Model		B	*β*	t	*p*	95% CI		VIF
						LL	UL	
1	(Constant)	20.727		8.577	<0.001	15.97	25.47	
	BC	2.703	0.218	3.584	<0.001	1.22	4.18	2.228
	AE	1.426	0.102	1.795	0.073	−0.13	2.98	1.959
	PA	−1.301	−0.102	−1.602	0.110	−2.89	0.29	2.449
	SS	3.564	0.314	5.047	<0.001	2.17	4.95	2.324
F (4, 469) = 33.218, *p* < 0.001, R = 0.470, R^2^ = 22.1% (adjusted R^2^ = 21.4%)								
2	(Constant)	24.725		7.513	<0.001	18.24	31.20	
	BC	2.473	0.199	3.243	0.001	0.97	3.97	2.234
	AE	1.278	0.092	1.608	0.108	−0.28	2.83	1.965
	PA	−1.202	−0.094	−1.481	0.139	−2.79	0.39	2.451
	SS	3.487	0.307	4.932	<0.001	2.09	4.87	2.325
	BMI (kg/m^2^)	−0.090	−0.050	−1.204	0.229	−0.23	0.05	1.010
F (5, 468) = 28.111, *p* < 0.001, R = 0.481, R^2^ = 23.1% (adjusted R^2^ = 22.3%)								
3	(Constant)	39.778		7.113	<0.001	28.80	50.75	
	BC	2.464	0.198	3.255	0.001	0.97	3.95	2.235
	AE	1.239	0.089	1.576	0.116	−0.30	2.78	1.966
	PA	−1.184	−0.093	−1.480	0.139	−2.75	0.39	2.451
	SS	3.445	0.304	4.939	<0.001	2.07	4.81	2.325
	BMI (kg/m^2^)	−0.079	−0.044	−1.066	0.287	−0.22	0.06	1.011
	Age	−0.304	−0.124	−3.007	0.003	−0.50	−0.10	1.035
F (6, 467) = 25.760, *p* < 0.001, R = 0.499, R^2^ = 24.9% (adjusted R^2^ = 23.9%)								
4	(Constant)	39.574		6.986	<0.001	28.44	50.70	
	BC	2.382	0.192	3.138	0.002	0.88	3.87	2.258
	AE	1.185	0.085	1.495	0.136	−0.37	2.74	2.002
	PA	−1.342	−0.105	−1.648	0.100	−2.94	0.26	2.457
	SS	3.407	0.300	4.847	<0.001	2.02	4.78	2.326
	BMI (kg/m^2^)	−0.096	−0.053	−1.292	0.197	−0.24	0.05	1.012
	Age	−0.299	−0.122	−2.946	0.003	−0.49	−0.09	1.038
	Education (secondary vs. primary/vocational)	0.225	0.013	0.169	0.866	−2.38	2.83	3.836
	Education (higher vs. primary/vocational)	1.818	0.109	1.367	0.172	−0.79	4.43	4.008
F (8, 465) = 20.279, *p* < 0.001, R = 0.509, R^2^ = 25.9% (adjusted R^2^ = 24.6%)								
5	(Constant)	40.326		7.047	<0.001	29.04	51.60	
	BC	2.387	0.193	3.133	0.002	0.89	3.88	2.375
	AE	1.166	0.084	1.460	0.145	−0.40	2.73	2.071
	PA	−1.381	−0.109	−1.733	0.084	−2.94	0.18	2.470
	SS	3.399	0.299	4.909	<0.001	2.04	4.75	2.331
	BMI (kg/m^2^)	−0.136	−0.076	−1.770	0.077	−0.28	0.01	1.164
	Age	−0.308	−0.127	−3.154	0.002	−0.50	−0.11	1.035
	Education (secondary vs. primary/vocational)	−0.082	−0.005	−0.062	0.951	−2.69	2.53	3.846
	Education (higher vs. primary/vocational)	1.539	0.092	1.146	0.252	−1.10	4.17	4.015
	Socioeconomic conditions (unsatisfactory)	−1.224	−0.062	−1.485	0.138	−2.84	0.39	1.092
F (9, 464) = 18.318, *p* < 0.001, R = 0.512, R2 = 26.2% (adjusted R^2^ = 24.8%)								

BC, Body Acceptance; AE, Appearance Evaluation; PA, Partner Acceptance; SS, Sexual Satisfaction; BMI, body mass index; B, unstandardized coefficient; *β*, standardized coefficient; CI, confidence interval; LL, lower limit; UL, upper limit; VIF, variance inflation factor. Reference categories: primary/vocational education and satisfactory socioeconomic conditions.

**Table 4:** Hierarchical Linear Regression Models Examining Factors Associated with Weight Concern Scores on the Body Esteem Scale from Subscales of the Scale of Assessment of Self-Attractiveness in a Relationship and Covariates.

Model		B	*β*	t	*p*	95% CI		VIF
						LL	UL	
1	(Constant)	15.276		5.049	<0.001	9.33	21.21	
	BC	2.707	0.234	3.725	<0.001	1.27	4.13	2.228
	AE	1.578	0.121	1.987	0.047	0.01	3.13	1.959
	PA	−1.194	−0.100	−1.439	0.151	−2.82	0.43	2.449
	SS	1.213	0.114	1.739	0.083	−0.15	2.58	2.324
F (4, 469) = 28.297, *p* < 0.001, R = 0.441, R^2^ = 19.4% (adjusted R^2^ = 18.8%)								
2	(Constant)	35.229		10.610	<0.001	28.71	41.74	
	BC	2.719	0.235	4.013	<0.001	1.38	4.05	2.234
	AE	1.459	0.112	1.930	0.054	−0.02	2.94	1.965
	PA	−1.009	−0.085	−1.322	0.187	−2.50	0.49	2.451
	SS	1.058	0.099	1.600	0.110	−0.24	2.35	2.325
	BMI (kg/m^2^)	−0.633	−0.369	−8.908	<0.001	−0.77	−0.49	1.010
F (5, 468) = 42.974, *p* < 0.001, R = 0.561, R^2^ = 31.5% (adjusted R^2^ = 30.7%)								
3	(Constant)	40.635		7.187	<0.001	29.52	51.74	
	BC	2.708	0.234	3.999	<0.001	1.37	4.04	2.235
	AE	1.453	0.112	1.922	0.055	−0.03	2.93	1.966
	PA	−1.005	−0.085	−1.315	0.189	−2.50	0.49	2.451
	SS	1.050	0.098	1.588	0.113	−0.25	2.35	2.325
	BMI (kg/m^2^)	−0.633	−0.368	−8.909	<0.001	−0.77	−0.49	1.011
	Age	−0.108	−0.046	−1.076	0.282	−0.30	0.08	1.035
F (6, 467) = 36.234, *p* < 0.001, R = 0.564, R^2^ = 31.8% (adjusted R^2^ = 30.9%)								
4	(Constant)	40.880		7.129	<0.001	29.63	52.12	
	BC	2.672	0.231	3.950	<0.001	1.34	4.00	2.258
	AE	1.417	0.109	1.873	0.062	−0.07	2.90	2.002
	PA	−1.051	−0.089	−1.374	0.170	−2.55	0.45	2.457
	SS	1.011	0.095	1.527	0.127	−0.28	2.31	2.326
	BMI (kg/m^2^)	−0.633	−0.368	−8.907	<0.001	−0.77	−0.49	1.012
	Age	−0.105	−0.045	−1.045	0.297	−0.30	0.09	1.038
	Education (secondary vs. primary/vocational)	−1.669	−0.112	−1.256	0.210	−4.28	0.94	3.836
	Education (higher vs. primary/vocational)	−0.714	−0.047	−0.536	0.592	−3.32	1.90	4.008
F (8, 465) = 27.791, *p* < 0.001, R = 0.569, R^2^ = 32.3% (adjusted R^2^ = 31.2%)								
5	(Constant)	41.601		7.184	<0.001	30.25	52.95	
	BC	2.683	0.225	3.822	<0.001	1.30	4.06	2.375
	AE	1.686	0.126	2.288	0.023	0.23	3.13	2.071
	PA	−1.256	−0.102	−1.709	0.088	−2.70	0.18	2.470
	SS	1.035	0.094	1.623	0.106	-0.21	2.28	2.331
	BMI (kg/m^2^)	−0.612	−0.356	−8.594	<0.001	−0.75	−0.47	1.164
	Age	−0.124	−0.053	−1.360	0.173	−0.30	0.05	1.035
	Education (secondary vs. primary/vocational)	−2.066	−0.126	−1.685	0.093	−4.47	0.34	3.846
	Education (higher vs. primary/vocational)	−1.134	−0.070	−0.916	0.360	−3.56	1.29	4.015
	Socioeconomic conditions (unsatisfactory)	−0.712	−0.037	−0.937	0.350	−2.20	0.78	1.092
F (9, 464) = 24.794, *p* < 0.001, R = 0.570, R^2^ = 32.5% (adjusted R^2^ = 31.2%)								

BC, Body Acceptance; AE, Appearance Evaluation; PA, Partner Acceptance; SS, Sexual Satisfaction; BMI, body mass index; B, unstandardized coefficient; *β*, standardized coefficient; CI, confidence interval; LL, lower limit; UL, upper limit; VIF, variance inflation factor. Reference categories: primary/vocational education and satisfactory socioeconomic conditions.

**Table 5:** Hierarchical Linear Regression Models Examining Factors Associated with Physical Condition Scores on the Body Esteem Scale from Subscales of the Scale of Assessment of Self-Attractiveness in a Relationship and Covariates.

Model		B	*β*	t	*p*	95% CI		VIF
						LL	UL	
1	(Constant)	16.718		6.648	<0.001	11.78	21.65	
	BC	2.265	0.230	3.516	<0.001	0.99	3.53	2.228
	AE	0.440	0.039	0.621	0.535	−0.95	1.83	1.959
	PA	−0.724	−0.071	−0.980	0.328	−2.17	0.72	2.449
	SS	1.422	0.157	2.322	0.021	0.21	2.62	2.324
F (4, 469) = 19.367, *p* < 0.001, R = 0.377, R^2^ = 14.2% (adjusted R^2^ = 13.4%)								
2	(Constant)	26.084		9.087	<0.001	20.44	31.72	
	BC	2.110	0.214	3.469	0.001	0.91	3.30	2.234
	AE	0.341	0.031	0.523	0.601	−0.95	1.63	1.965
	PA	−0.631	−0.062	−0.927	0.354	−1.96	0.70	2.451
	SS	1.301	0.144	2.171	0.030	0.12	2.47	2.325
	BMI (kg/m^2^)	−0.320	−0.257	−5.469	<0.001	−0.43	−0.20	1.010
F (5, 468) = 22.354, *p* < 0.001, R = 0.439, R^2^ = 19.3% (adjusted R^2^ = 18.4%)								
3	(Constant)	31.082		6.393	<0.001	21.52	40.64	
	BC	2.109	0.214	3.483	0.001	0.92	3.29	2.235
	AE	0.337	0.030	0.518	0.605	−0.95	1.63	1.966
	PA	−0.629	−0.062	−0.925	0.355	−1.96	0.70	2.451
	SS	1.296	0.143	2.157	0.032	0.11	2.47	2.325
	BMI (kg/m^2^)	−0.320	−0.257	−5.477	<0.001	−0.43	−0.20	1.011
	Age	−0.254	−0.123	−2.814	0.005	−0.43	−0.07	1.035
F (6, 467) = 20.929, *p* < 0.001, R = 0.460, R^2^ = 21.2% (adjusted R^2^ = 20.2%)								
4	(Constant)	30.969		6.270	<0.001	21.24	40.69	
	BC	2.019	0.205	3.318	0.001	0.82	3.21	2.258
	AE	0.299	0.027	0.458	0.647	−0.99	1.59	2.002
	PA	−0.680	−0.067	−0.996	0.320	−2.01	0.65	2.457
	SS	1.243	0.137	2.063	0.040	0.06	2.42	2.326
	BMI (kg/m^2^)	−0.322	−0.258	−5.486	<0.001	−0.43	−0.20	1.012
	Age	−0.253	−0.123	−2.800	0.005	−0.43	−0.07	1.038
	Education (secondary vs. primary/vocational)	−0.751	−0.056	−0.562	0.574	−3.36	1.86	3.836
	Education (higher vs. primary/vocational)	0.347	0.026	0.260	0.795	−2.26	2.96	4.008
F (8, 465) = 15.951, *p* < 0.001, R = 0.464, R^2^ = 21.5% (adjusted R^2^ = 20.2%)								
5	(Constant)	31.650		6.342	<0.001	21.84	41.45	
	BC	2.032	0.206	3.266	0.001	0.80	3.25	2.375
	AE	0.620	0.056	0.949	0.343	−0.66	1.90	2.071
	PA	−0.949	−0.094	−1.458	0.146	−2.22	0.33	2.470
	SS	1.288	0.143	2.280	0.023	0.17	2.39	2.331
	BMI (kg/m^2^)	−0.297	−0.209	−4.707	<0.001	−0.42	−0.17	1.164
	Age	−0.263	−0.137	−3.284	0.001	−0.42	−0.10	1.035
	Education (secondary vs. primary/vocational)	−0.253	−0.019	−0.233	0.816	−2.38	1.88	3.846
	Education (higher vs. primary/vocational)	0.476	0.036	0.434	0.664	−1.68	2.63	4.015
	Socioeconomic conditions (unsatisfactory)	−1.046	−0.067	−1.554	0.121	−2.37	0.27	1.092
F (9, 464) = 14.490, *p* < 0.001, R = 0.468, R^2^ = 21.9% (adjusted R^2^ = 20.4%)								

BC, Body Acceptance; AE, Appearance Evaluation; PA, Partner Acceptance; SS, Sexual Satisfaction; BMI, body mass index; B, unstandardized coefficient; *β*, standardized coefficient; CI, confidence interval; LL, lower limit; UL, upper limit; VIF, variance inflation factor. Reference categories: primary/vocational education and satisfactory socioeconomic conditions.

## Data Availability

The data presented in this study are available upon request from the corresponding author.

## Abbreviations

The following abbreviations are used in this manuscript:

BES	Body Esteem Scale
BMI	Body Mass Index
CI	Confidence Interval
FMP	Final Menstrual Period
SAAR	Scale of Assessment of Self-Attractiveness in a Relationship
SD	Standard Deviation
STRAW + 10	Stages of Reproductive Aging Workshop +10
STROBE	Strengthening the Reporting of Observational Studies in Epidemiology
VIF	Variance Inflation Factor
